# New Avenues to Design *Toxoplasma* Vaccines Based on Oocysts and Cysts

**DOI:** 10.3389/fimmu.2022.910961

**Published:** 2022-06-06

**Authors:** David Arranz-Solís, Jeroen P. J. Saeij

**Affiliations:** Pathology, Microbiology and Immunology department, School of Veterinary Medicine, University of California Davis, Davis, CA, United States

**Keywords:** *Toxoplasma*, CRISPR/Cas9, oocysts, cysts, sporozoites, vaccine, cats

## Abstract

Toxoplasmosis is a worldwide disease affecting all warm-blooded animals, including humans. Vaccination strategies aimed at inducing an efficient immune response while preventing transmission have been attempted in the past. While many different approaches can partially protect immunized animals against subsequent infections, full and lasting protection is rarely attained and only with live-attenuated vaccines. In addition, vaccines based on mutant strains that are deficient in forming the chronic phase of the parasite (such as Toxovax™) cannot be extensively used due to their zoonotic potential and the possibility of reversion to virulent phenotypes. An increasing number of studies using emerging genetic-engineering tools have been conducted to design novel vaccines based on recombinant proteins, DNA or delivery systems such as nanoparticles. However, these are usually less efficient due to their antigenic simplicity. In this perspective article we discuss potential target genes and novel strategies to generate live-attenuated long-lasting vaccines based on tissue cysts and oocysts, which are the environmentally resistant chronic forms of *Toxoplasma*. By selectively disrupting genes important for parasite dissemination, cyst formation and/or sporozoite invasion, alone or in combination, a vaccine based on a live-attenuated strain that elicits a protective immune response while preventing the transmission of *Toxoplasma* could be created. Finally, further improvements of protocols to generate *Toxoplasma* sexual stages *in vitro* might lead to the production of oocysts from such a strain without the need for using mice or cats.

## Introduction

The phylum *Apicomplexa* comprises a large group of protozoan parasites, many of which cause disease in humans and livestock, such as *Plasmodium* spp., *Toxoplasma gondii*, *Neospora* spp. or *Eimeria* spp (1). Toxoplasmosis is a worldwide zoonotic disease and one of the leading causes of foodborne illness in the USA. It can affect virtually all mammals and birds, including approximately one third of humans (2). Although the majority of infected healthy individuals are asymptomatic, this disease can cause neonatal mortality, abortions and a wide variety of neurological symptoms, especially in immunocompromised or congenitally infected patients (3, 4). In addition, toxoplasmosis causes important economic losses in the livestock sector related to reproductive failure, mainly in sheep and goats (5).

In intermediate hosts, which include any warm-blooded animal, infection can occur after ingestion of tissue cysts present in meat or viscera from infected animals, or oocysts shed in the feces of an infected feline (definitive hosts that can also act as intermediate hosts). Upon ingestion, these orally infectious forms readily differentiate into tachyzoites, the fast-replicating stage responsible for proliferation and dissemination throughout the host and also for the clinical symptoms during the acute phase of the disease. After eliciting an immune response, tachyzoites transform into bradyzoites, the slow-replicating stage that forms tissue cysts. These are found mainly in the brain and muscle, remaining intact for long periods, possibly throughout the life of the host (6). When a cat ingests tissues from an infected animal, bradyzoites invade intestinal epithelial cells and differentiate into merozoites. After multiple replication cycles, those merozoites eventually differentiate into micro- (male) and macro- (female) gametes that upon fusion will ultimately form hundreds of millions of oocysts, which are shed unsporulated within cat feces (7). Once in the environment, and under favorable conditions of humidity, aeration and temperature, sporulation takes place and another asexual form of the parasite, called sporozoites, develops within oocysts. The mature (or sporulated) *Toxoplasma* oocyst has in its final form 2 sporocysts containing 4 sporozoites each. Oocysts are highly stable in the environment, extremely resistant to inactivation procedures, and exceptionally infectious to intermediate hosts, as even a single oocyst is capable of eliciting the infection. The oocyst and sporocyst walls provide an important protective barrier and likely protect the sporozoites from environmental stressors (8). For example, many studies have described that *Toxoplasma* oocysts can withstand freezing conditions for weeks and temperatures above 50 °C for several minutes (9–13). Similarly, many chemicals and common disinfectants, even strong solutions such as household bleach, ethanol or formalin, among others, are not able to inactivate oocysts (11, 14–18).

## Toxoplasmosis in Cats

*Toxoplasma* infections in cats are typically subclinical. Nevertheless, congenitally infected kittens are the most likely to have symptoms, although healthy adult cats may also be affected (7, 19). The most common clinical signs of toxoplasmosis in cats include fever, diarrhea, or other severe signs such as pneumonia, ocular disease and, importantly, neurological signs. This concern is not to be disregarded, as the worldwide estimated seroprevalence for *Toxoplasma* in domestic cats (*Felis catus*) is 35–40%, being even higher in wild felids (~60-65%) (20, 21). In the USA, local seroprevalence studies have reported figures ranging from 16% to 43% (22, 23). However, these *Toxoplasma* seroprevalence rates might be overestimated as these studies employed serological assays with total *Toxoplasma* lysates as antigens, which could cross-react with other closely related parasites such as *Cystoisospora* spp., *Hammondia* spp. or *Sarcocystis* spp (24). Hence, future approaches using more specific antigens, such as recombinant proteins or peptides, could provide more accurate estimates (25, 26).

Although it is commonly assumed that cats only shed oocysts the first time they get infected and only for a short period of time, a growing body of evidence suggests that cats might shed oocysts more than once if they become immunosuppressed (27, 28), infected with other coccidian parasites such as *Cystoisospora* (29) or with heterologous *Toxoplasma* strains (30, 31). This, together with the extraordinary infectivity and durability of oocysts, supports the idea that cats play a critical role in the epidemiology of toxoplasmosis. Therefore, if cats could be vaccinated so they no longer shed infectious oocysts, this would drastically reduce the exposure of humans and animals. Indeed, a recent report evaluated the effects of a hypothetical cat vaccine on reducing the presence of *Toxoplasma* oocysts in the environment and its implication in human infections (32). Although it was deemed unfeasible to obtain a complete elimination of *Toxoplasma* oocyst-originated transmission in large populations, vaccinating cats could still significantly decrease the presence of oocysts in the environment and thus the probability of humans getting infected through contaminated food or water. In addition, it would also decrease the percentage of animals infected and therefore the economic losses and presence of tissue cysts in their meat, which would in turn lessen human infection through consumption of animal products.

## Vaccines in Animals

Currently, there is only one *Toxoplasma* vaccine commercially available, and it is only authorized for use on sheep (33). This live attenuated vaccine (Toxovax™) is based on tachyzoites from the S48 strain, which is unable to form cysts or oocysts due to its exceptionally prolonged *in vitro* manipulation (34). Even though this vaccine can partially protect immunized sheep against abortion, it presents critical shortcomings in terms of safety, production and stability. Since the genetic basis for its attenuation is unknown, a possible reversion to virulence cannot be dismissed. Moreover, the short viability of tachyzoites in an extracellular environment greatly hinders its production and maintenance in the long term. When used in cats, the S48 strain elicited a strong immune response, while cats did not produce oocysts (35). Similarly, the chemically-induced mutant strain T-263, which lost its ability to form oocysts (36), was also tested in cats and shown to prevent oocyst shedding after subsequent challenges (37, 38). This deficiency was recently ascribed to either a deficient fertilization or a blockage of oocyst wall formation, as schizonts and gamonts were observed in the intestine, albeit without generation of oocysts (39). However, akin to the “cyst-less” S48 strain, the gene(s) affected by the chemical mutagenesis of the “oocyst-less” T-263 strain are not known (36). It seems that to achieve immunity to oocyst shedding, it is needed that bradyzoites invade intestinal epithelial cells and convert to merozoites, as trials with T-263 tachyzoites directly delivered into the duodenum of cats did not confer protection against subsequent infections, despite eliciting a marked antibody response. Conversely, oral administration of cysts or bradyzoites prevented oocyst shedding after a heterologous challenge (38). In another field trial, resident cats from a pig farm in Illinois, USA, were orally administered with frozen T-263 bradyzoites and a decreased seroprevalence in the farmed pigs was observed in the following 3 years, suggesting a lower oocyst environmental contamination (40). Other vaccination trials in cats with mutant *Toxoplasma* strains have been attempted in the past, most of which elicited protection against subsequent infections (35). For instance, vaccination of cats with the non-persistent and temperature-sensitive TS-4 RH strain (36) did not induce oocyst shedding (41). Similarly, a Beverly strain that was modified by irradiation treatments was able to partially prevent cats from shedding oocysts (42). However, akin to the S48 and T-263 strains, the exact gene modifications of these mutant strains are not known, thus rendering them not reliable for its usage in animals, especially those with the virulent RH background.

### New Strategies for *Toxoplasma* Vaccination

If a parasite similar to S48 or T-263 unable to generate orally infectious forms (tissue cysts and/or oocysts) could be engineered with alterations in known genes, the safety problem could be partially solved. For example, in a recent study, a mutant strain deficient in the microgamete gene HAP2 was used to infect cats, and only a very reduced number of misshapen oocysts that failed to sporulate were produced (43). Although vaccination of cats with this strain prevented oocyst shedding after a subsequent challenge with the CZ strain, it did not preclude systemic dissemination of the parasite and cyst formation in their brains (43). Another example of a mutant strain for which the gene modification is known that was tested in cats is the MIC1-3 KO strain (44). Nevertheless, this mutant parasite from a type I background was not able to prevent cats from shedding oocysts when tachyzoites were used either subcutaneously or by oral route, a fact that reinforces the aforementioned notion that bradyzoite invasion of the intestinal cells is needed to confer protection against oocyst formation in vaccinated cats. Regardless, and similar to the S48 or T-263 strain, the production of these vaccines would face similar drawbacks in terms of stability, as tachyzoites would be needed to infect mice and obtain tissue cysts for oral vaccination. By contrast, vaccines based on oocysts would allow long-term conservation of the formulations on account of their durability and stability, facilitating production and maintenance. In addition, these oocysts could have the potential to be devised as a vehicle to deliver or express antigenic proteins from other parasites (e.g., *Neospora caninum* to protect dogs from shedding *Neospora* oocysts). Because bradyzoites are critical components for the transmission of infection, the use of a vaccine capable of inducing a protective immune response while preventing conversion to the bradyzoite stage would represent an ideal approach to reduce the presence of tissue cysts in the meat of infected animals, decreasing in turn human exposure. Therefore, the development of a novel vaccine consisting of *Toxoplasma* oocysts from a strain that can no longer convert into bradyzoites in the intermediate hosts could serve as a valuable tool. However, there is a challenge to generate the oocysts from a bradyzoite-deficient mutant strain, since a parasite is needed that can complete the sexual cycle in cats but from which the oocysts can no longer convert into tissue cysts upon ingestion by intermediate hosts.

By using the CRISPR/Cas9 technology, a strain defective in genes coding for proteins involved in tissue cyst formation can be obtained. Notwithstanding, this defect should only start once the parasites are in the cat intestine. To accomplish this, Cas9 could be expressed from the promoter of a *Toxoplasma* gene that is only expressed in the intestinal stages, which would allow disruption of genes essential for tissue cyst formation only when *Toxoplasma* undergoes the sexual cycle in cats. Different strategies, such as having the bradyzoite genes of interest flanked by LoxP sites and the CRE recombinase expression driven by specific intestinal-stage promoters could also be devised to attain the same goal. A number of genes have been shown to be active only in the intestine of the cat (43, 45). Among them, the RNA expression of two SRSs (SAG1-Related Surface) proteins, SRS22B and SRS22H, as well as the megakaryocyte stimulating factor (MSF), was highly upregulated in *Toxoplasma* cat intestinal stages compared to bradyzoites. Hence, by inserting the promoters of these genes upstream of the Cas9 (or CRE) coding sequence, the endonuclease (or recombinase) should be only expressed when the parasites convert into merozoites in the intestine of cats, while it should not in tachyzoites or bradyzoites.

Different research groups have identified molecular mediators that regulate the conversion of tachyzoites to bradyzoites and cyst formation. These include the specific cyst wall glycoprotein (CST1), which is a structural component of the cyst wall (46), the nucleotide-sugar transporter 1 (NST1), necessary for the glycosylation of the cyst wall (47), or the Apetala-2 transcription factor AP2XI-4, which is important for bradyzoite gene expression during conversion and cyst formation (48). Nevertheless, the most remarkable advancement in the understanding of the tachyzoite-to-bradyzoite conversion was recently made by Waldman et al., when the master regulator of bradyzoite formation was described (49). The Bradyzoite-Formation Deficient 1 (BFD1) protein was shown to be essential for bradyzoite conversion: its ablation rendered parasites unable to form tissue cysts both *in vitro* and *in vivo*, while its conditional overexpression is sufficient to induce differentiation (49).

Notwithstanding, when Cas9 is activated in the intestine of the cat, and in the absence of a selection process as it would happen *in vitro*, it is likely that not every single parasite will get the targeted genes of interest disrupted, as the double strand breaks can be repaired with small indels and retain the function of the gene. Because of this, introducing several gRNAs for different genes will increase the chance of having at least one or more of the targeted genes disrupted in each parasite, thus decreasing the likelihood of having intact or wild-type parasites after going through a cat. To overcome this possibility other approaches can also be adopted. For instance, targeting genes important for the dissemination/migration of the parasite, such as ROP17 (50) or the recently described TgWIP (51), would also considerably hinder the ability of *Toxoplasma* to reach the brain where the majority of cysts are formed. Likewise, if a gene that is important for the *in vivo* fitness of the parasite, such as the dense granule Myc regulation protein (MYR)1 (52), is targeted, it would add additional layers to ensure parasites do not form tissue cysts. By making a parasite strain defective in one or more of the aforementioned genes, it is likely that viable tissue cysts are no longer formed, while its *in vitro* fitness remains unaltered. If the generation of oocysts from this mutant strain could be engineered, intermediate hosts could be orally vaccinated. In this case, sporozoites would invade intestinal cells, and eventually convert into susceptible tachyzoites with compromised dissemination and virulence, and unable to differentiate into bradyzoites. Therefore, and akin to a natural infection, these oocysts should elicit a protective mucosal and systemic immune response. However, as opposed to a natural infection where bradyzoites and tissue cyst would be formed, parasites will be eventually eliminated by the immune response without the possibility to form orally infectious tissue cysts.

Although oocysts are extremely resistant and long-lasting, and a single cat would be sufficient to produce enough oocysts (hundreds of millions) for a theoretical large-scale vaccination test, growing ethical concerns with animal experimentation warrants future endeavors to find alternative methods to produce these oocysts. A recent breakthrough study by Di Genova et al. showed that the natural lack of the enzyme Δ-6 desaturase in felines, which does not occur in any other mammal, causes accumulation of linoleic acid in their intestine, cueing *Toxoplasma* to initiate its sexual reproduction (53). By exploiting this feline’s unique trait, they made it possible for the first time to produce *Toxoplasma* sexual stages and oocysts *in vitro* by culturing cat and mouse intestinal organoids infected with *Toxoplasma* in the presence of linoleic acid and a Δ-6 desaturase inhibitor. Moreover, the administration of these two compounds in mice orally infected with tissue cysts was sufficient to trigger oocyst shedding in their feces. However, both the yields and sporulation capacity of the oocysts obtained by these methods showed important deficiencies compared to those naturally produced in cats (53). Another recent discovery made by Farhat et al. (54) described the ATPase microrchidia protein (MORC), which blocks gene accessibility by its association with the histone deacetylase HDAC3 and several AP2 transcription factors. By acting as a repressor, it has been suggested that MORC could be a master regulator of developmental directionality, blocking the expression of non-tachyzoite genes. Indeed, depletion of MORC triggered bradyzoite conversion and the expression of several intestinal-stage specific genes, including genes encoding macrogamete and microgamete, oocyst wall, and sporozoite-specific proteins, among others (54). Therefore, MORC could possibly be exploited to induce sexual development without the need for experimental infections in cats and directly from tachyzoites. Finally, a novel avenue that could help to improve the *in vitro* production of oocysts could be the recently described micro-physiological system of intestinal tissue described by Humayun et al. (55). By using a novel micromolding technique to generate hollow structures, artificial tubes of intestine can be engineered, recapitulating the lumen geometries of the gut (55). If this system were to be combined with either, or both, of the discoveries mentioned above, the culture of *Toxoplasma* intestinal stages *in vitro* mirroring natural conditions could be significantly improved. In summary, although a refinement of these models is needed, they lead the way towards a future approach where a fully developed and standardized system can be broadly used to produce oocysts with similar features to those from cats without having to resort to *in vivo* assays.

### Vaccines in the Definitive Host, the Cat

In contrast to intermediate hosts, to which oocysts are extremely infective, cats are much more likely to become infected, and subsequently shed oocysts, following ingestion of tissue cysts rather than tachyzoites or oocysts (56, 57). Indeed, the ingestion of one bradyzoite is usually sufficient to induce feline infection and oocyst shedding within the first week post-infection, whereas a feline usually needs to ingest 1000 oocysts to develop a less efficient infection that will lead to a decreased and delayed shedding of oocysts (56, 57). This difference is probably due to the fact that following the ingestion of tissue cysts, some bradyzoites convert to tachyzoites and some to schizonts (merozoites), which replicate asexually in the intestinal tissue before beginning sexual reproduction (58). On the other hand, when cats are orally infected with oocysts, the released sporozoites cannot start the sexual cycle in the gut of the cat; instead, they differentiate into the fast-dividing tachyzoite stage that subsequently converts into the bradyzoite stage, which only then can differentiate into intestinal stages (56, 59). This is demonstrated by the fact that the oocyst-shedding prepatent period greatly varies when cats get infected with cysts (usually less than one week) or oocysts (more than 2-3 weeks) (56, 58, 59). Because the hypothetical vaccine described above will no longer be able to form bradyzoites, it would be expected that cats orally infected with oocysts from such a strain will not shed oocysts, as intestinal stages cannot be formed in the absence of bradyzoites. Notwithstanding, this has never been categorically proven, and it cannot be ruled out that some epigenetic conditions in the intestine might trigger *Toxoplasma* differentiation into merozoites without having to go through the bradyzoite stage.

**Figure 1:**
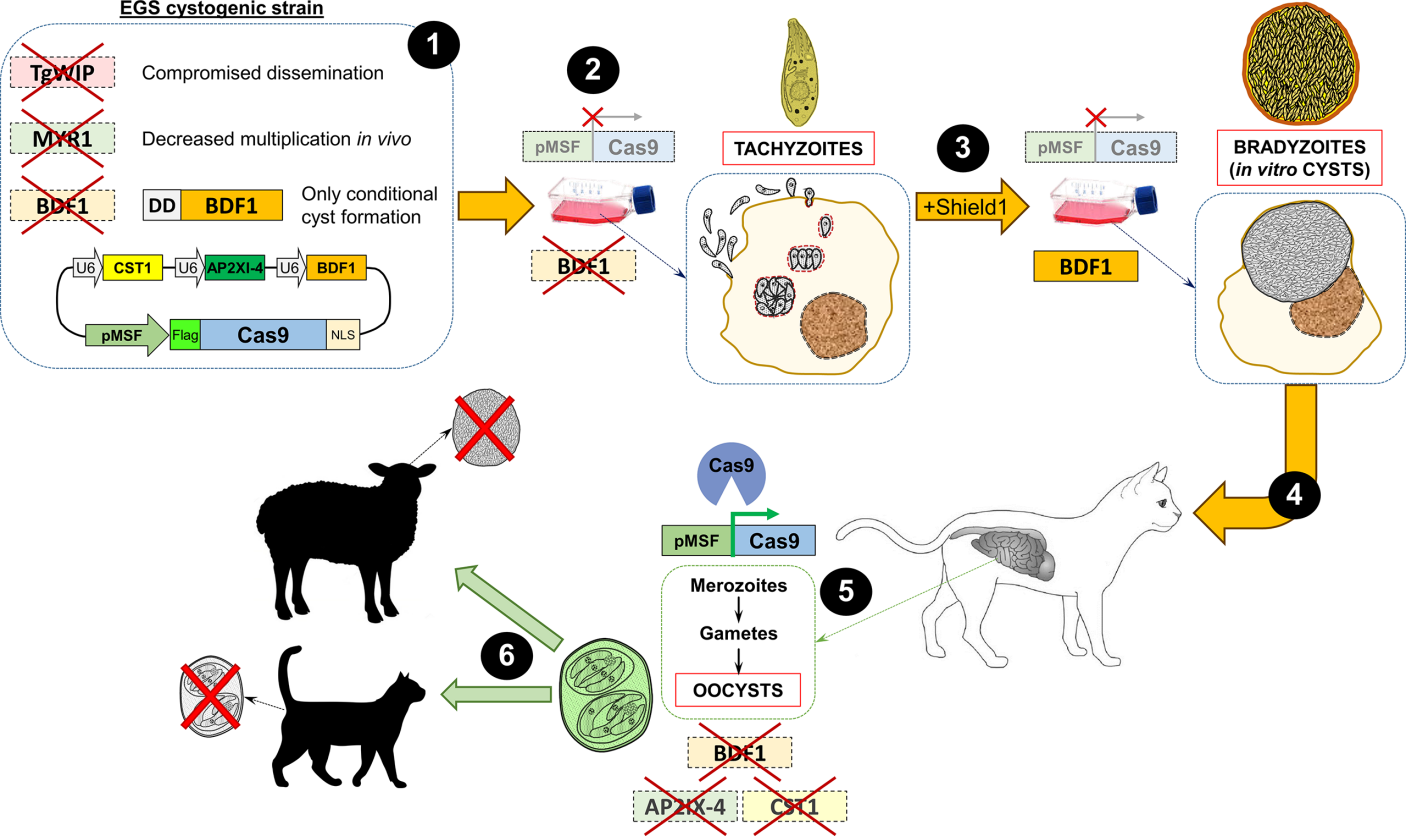
Layout of one of the possible approaches to devise a hypothetical oocyst-based ideal vaccine to prevent transmission in *Toxoplasma*. (1) A cystogenic strain, such as EGS, can be used as the parental strain to disrupt genes important for the dissemination of tachyzoites (*Tg*WIP), *in vivo* fitness (MYR1), and bradyzoite conversion (BDF1). In addition, a construction in which an exogenous copy of BDF1 is conditionally regulated with the destabilizing domain (DD) can be inserted so that cyst formation can only be obtained in the presence of Shield-1. Finally, a plasmid containing a merozoite promoter (in this example the megakaryocyte stimulating factor -MSF-) driving the expression of the endonuclease Cas9 (epitope-tagged, in the example, with Flag) and several single guide (sg)RNAs cassettes targeting genes important for bradyzoite conversion and cyst formation, such as CST1, AP2XI-4 and BDF1, is also included. The latter is included as an additional safety measure in the unlikely scenario that the BDF1 exogenous copy introduced becomes active or leaky in the future. (2) Due to the presence of the merozoite promoter, the endonuclease Cas9 is not expressed in the tachyzoite stage. Moreover, since all the aforementioned targeted genes are dispensable *in vitro*, parasites can be grown normally in tissue culture. Finally, despite being a cystogenic strain, in the absence of Shield-1, the DD domain degrades BDF1 thus preventing tachyzoite-to-bradyzoite conversion (49). (3) Upon addition of Shield-1 to the culture media, BDF1 is constitutively expressed, strongly eliciting the conversion to the bradyzoite stage and cyst formation *in vitro*, while Cas9 is still not expressed. (4) Cysts obtained *in vitro* from the mutant EGS strain can be used to orally infect cats (64). (5) In the intestine of the cat, Cas9 is finally expressed and can be directed to knock out several genes involved in the tachyzoite-to-bradyzoite conversion, using specific guide RNA sequences present in the construction (see step 1). (6) Finally, the oocysts shed in the feces of the infected cat will be used to vaccinate other animals to elicit a protective immune response while preventing the parasite from converting to bradyzoites and forming tissue cysts. In the case of cats, this would also theoretically mean that intestinal stages will not be formed, as these can only be formed from bradyzoites, and thus oocysts will not be shed in the feces. U6: RNA polymerase III promoter; NLS: nuclear localization signal.

Regardless, it is possible that for the definitive hosts a bradyzoite-based vaccine would be more efficient. However, to obtain bradyzoites from a cyst-less strain, a conditional disruption of cyst-essential genes is needed. For example, by knocking out the *BFD1* gene and replacing the endogenous locus with a regulatable BDF1 version, bradyzoite conversion could be tightly controlled (49). To achieve this goal, the expression of the *BFD1* gene can be conditionally regulated by using the Tetracycline(Tet)-ON (60, 61) or the FKBP-derived destabilization domain (DD) system (49, 62). This way, by the addition of Tetracycline or the Shield-1 ligand, respectively, bradyzoites can be transiently formed and cysts obtained to feed cats orally. After infection, and without the presence of Tetracycline or Shield-1, the strain will not be able to form bradyzoites anymore. However, the formation of oocysts in the intestine after infection with such a strain cannot be avoided. To circumvent this shortcoming and prevent formation of infective oocysts, a gene essential for gamete formation, such as the aforementioned microgamete-specific HAP2 gene (43), could be disrupted. In addition, the bradyzoite-containing cysts needed for oral infection of cats could be obtained from the EGS strain, which has been shown to undergo high levels of spontaneous cyst conversion under regular conditions *in vitro* (63). Furthermore, these *in vitro*-derived EGS cysts were orally infective to cats, which produced oocysts in their feces (64). It could therefore be an interesting avenue to use this strain, or other highly cystogenic strain, as a parental parasite for the generation of the genetically modified vaccine described above, so that cats can be administered orally with *in vitro* cysts without the need to infect mice. In this sense, it could further help to use an *in vitro* system that generates fully functional orally infectious tissue cysts, such as the recently described human myotube-based *in vitro* culture model (65). Overall, if a cat can be vaccinated with cysts conditionally obtained *in vitro* from a strain that is not able to form sexual stages in the intestine nor convert into bradyzoites anymore, a protective immune response will be elicited in the cats while transmission is halted. A possible approach to design a hypothetical ideal vaccine based on oocysts is shown in **Figure 1**.

## A Vaccine Based on Sporozoites

Despite being a critical stage for the transmission of *Toxoplasma*, sporozoites inside oocysts are under-studied, as they are not cultivatable *in vitro* and *in vivo* assays are restricted to a few laboratories with the resources to house cats. In contrast to tachyzoites, sporozoites within oocysts can stay viable outside a host for years even under harsh conditions. In addition, the oocyst and sporocyst walls provide an important protective barrier for sporozoites from environmental stressors. Therefore, a better understanding of the genes involved in extracellular survival and invasion of sporozoites present in oocysts could lead to the discovery of novel drug or vaccine targets that may prevent livestock or humans from getting infected by sporozoites. Furthermore, if the genetic basis for the extreme environmental resistance of sporozoites was known, it might be possible to exploit this to make other life stages, such as tachyzoites or other parasites, more viable extracellularly, which could enhance the shelf life of vaccines based on live parasites. Nonetheless, it is worth noting that this could lead to a potential misusage by creating *Toxoplasma* or other parasite mutant strains that are unnaturally resistant, constituting an ethical quandary (the recently coined “dual-use problem” term (66). However, this possibility exists for any *Toxoplasma* strain that is, for example, expressing a toxic heterologous protein, or using oocysts from any mutant strain. In this case, since the purpose is to use a recombinant strain deficient in dissemination with a deficient *in vivo* fitness, it would be unlikely that it can become more dangerous than a wild-type strain, even if tachyzoites were artificially made more viable extracellularly. Regardless, to avoid a morally undesirable usage, such hypothetical strains should be strictly contained and shared only under very specific and justified conditions. Moreover, it is also possible that transforming other life stages to make them more resistant by using genes related to environmental resistance could render them deficient in other aspects such as replication or undermine their natural behavior; for instance, it could be possible that tachyzoites would not be able to multiply at their usual fast pace anymore.

Be that as it may, we hypothesize that genes governing the resistance and infectivity of the sporozoite stage are specifically upregulated in sporulated oocysts compared to unsporulated oocysts. To identify such genes, we used data from a number of studies that examined the transcriptome and proteome of different *Toxoplasma* developmental stages, which are available on ToxoDB (67). We first identified 94 *Toxoplasma* proteins that had at least 10 unique peptides detected in at least one of the two proteomic analyses of oocysts but no peptides in any other stages (68, 69). We subsequently refined this list by identifying 34 *Toxoplasma* genes that are on average at least 4-fold upregulated in sporulated oocysts (day 4 and 10 after shedding) *vs.* unsporulated oocysts (day 0 after shedding) and *vs.* tachyzoites, bradyzoites and merozoites (43, 70, 71). We noted that among the highest expressed genes (**Table 1**) there are 4 genes that encode for putative late embryogenesis abundant proteins (LEAs). LEA proteins have been described in several organisms, including plants, invertebrates, and microorganisms, with a commonly ascribed role in resistance to environmental stresses such as drought, high salinity and freezing (72). Their abundance in sporozoites suggests that LEA proteins may be a critical component to sporozoites. One of the LEA proteins (TGME49_276850 also named TgERP -Embryogenesis-Related Protein-) was used in a serological assay and shown to be able to distinguish infections caused by ingestion of oocysts *vs*. tissue cysts (73). Some other interesting genes from our search included a DNA photolyase (TGME49_206400) that could be involved in DNA-repair mechanisms caused by UV light (74, 75) and a putative glutaredoxin-like protein (TGME49_227100) that could be related to oxidation repair enzyme processes (76, 77).

**Table 1:** Highly upregulated sporozoite genes candidate list.

Gene ID	Product Description	Other Information, Homologies, and Domains	OO4	OO10	OO0	MZ*	TZ	BZ
**TGME49_203890**	hypothetical protein		358	201	31	4	0	0
**TGME49_206400**	FAD binding domain of DNA photolyase domain-containing protein		1361	642	101	17	1	1
**TGME49_209920**	PAN domain-containing protein	MIC4L	965	237	0	1	0	4
**TGME49_214570**	hypothetical protein	Nuclear pore glycoprotein domain	370	300	20	0	0	1
**TGME49_220280**	mucin family glycoprotein		240	157	0	1	4	3
**TGME49_223430**	hypothetical protein		148	23	0	2	1	14
**TGME49_226230**	hypothetical protein		55	42	0	0	0	0
**TGME49_227100**	hypothetical protein	Putative Glutaredoxin	12391	7405	82	30	1	76
**TGME49_258200**	hypothetical protein	Putative glutamic acid rich protein	206	166	0	1	1	3
**TGME49_258550**	SAG-related sequence SRS28	SporoSAG	14087	3744	3	3	5	4
**TGME49_258810**	SAG-related sequence SRS27B		373	117	0	4	0	26
**TGME49_259670**	von Willebrand factor type A domain-containing protein	CTRP (CS protein-TRAP-related protein)	4595	1270	0	4	0	0
**TGME49_265120**	rhoptry neck protein, putative	SporoRON2	70	50	0	1	1	2
**TGME49_269380**	hypothetical protein	Elongation factor 4 and GTP-binding protein domains	84	74	0	0	1	0
**TGME49_270950**	hypothetical protein	Thymidylate kinase domain	761	709	6	2	2	1
**TGME49_271210**	4-alpha-glucanotransferase		782	139	0	20	0	1
**TGME49_272240**	Toxoplasma gondii family D protein		584	458	1	0	0	0
**TGME49_273510**	hypothetical protein		369	243	0	0	0	2
**TGME49_276850**	hypothetical protein	LEA-TgERP (Embryogenesis related protein)	11753	3640	1	1	0	1
**TGME49_276860**	hypothetical protein	LEA (Late Embryogenesis Abundant protein)	282	203	1	1	0	1
**TGME49_276870**	hypothetical protein	LEA (Late Embryogenesis Abundant protein)	2354	499	23	1	1	1
**TGME49_276880**	hypothetical protein	LEA (Late Embryogenesis Abundant protein)	4446	1240	5	0	0	1
**TGME49_278120**	SCP family extracellular subfamily protein		531	326	0	3	1	1
**TGME49_281590**	hypothetical protein	Putative periplasmic substrate binding protein	119626	15174	5	2	2	13
**TGME49_292350**	hypothetical protein	Putative *Toxoplasma* Family A-likely surface antigen	863	239	1	0	0	2
**TGME49_293620**	hypothetical protein		48	26	0	0	0	2
**TGME49_294600**	Toxoplasma gondii family D protein	Alpha-2U-globulin conserved domain	3772	3004	7	5	0	1
**TGME49_297280**	hypothetical protein		828	142	0	3	0	19
**TGME49_309540**	hypothetical protein		69	57	1	0	0	1
**TGME49_315260**	alanine dehydrogenase		185	330	33	36	11	14
**TGME49_315730**	apical membrane antigen 1 protein	SporoAMA1	1247	357	68	27	0	35
**TGME49_319890**	hypothetical protein	Putative Tyrosine-rich protein	38312	6594	0	2	10	3
**TGME49_320280**	hypothetical protein	Hemin storage protein, chitinase and glycosyl hydrolase domains	85	68	0	0	0	1
**TGME49_320530**	hypothetical protein	Putative Tyrosine-rich protein	6656	4086	2	0	0	0

Indicated are the gene IDs for which at least 10 unique peptides were detected in proteomic analysis of oocysts (59, 60) and 0 peptides in any other stages, and that had at least 4-fold higher expression in sporulated (day 4 and day 10 –OO4 and OO10-) vs. unsporulated oocysts (day 0 -OO0-) (70) and vs. tachyzoites (TZ), bradyzoites (BZ), and merozoites (MZ) (43). Numbers represent the average expression level in transcripts per million (TPM). *For the merozoite value, the average of the 5 Enteroepithelial stages (EES) described by Ramakrishnan et al. was used (43)

Moreover, multiple genes in **Table 1** are predicted to be involved in sporozoite attachment/invasion. For example, Sporo-Surface Antigen (SAG), also called SAG1-related Sequence (SRS)28, is predicted to be involved in parasite attachment to negatively charged host surface molecules such as glycosaminoglycans (78), and tachyzoites overexpressing SporoSAG have enhanced invasion in HFFs (79). SporoAMA1 and SporoRON2 have been shown to be involved in parasite invasion, as the addition of recombinant sporoRON2-domain 3, which competes with the SporoAMA1-sporoRON2 interaction, significantly inhibited sporozoite invasion (79). However, *Toxoplasma* has 4 *AMA-1* like genes and 3 *RON2*-like genes, combinations of which are differentially expressed in different life stages. Therefore, other AMA-RON pairs can compensate for the absence of specific pairs (80) and, besides sporoSAG, it is predicted that sporozoites express at least another 10 SRS proteins. Hence, it is unlikely that only one is sufficient to prevent sporozoite invasion, which would mean that immunization with a SRS, sporoAMA or sporoRON2 based vaccine might not be successful. TGME49_259670 has significant homology to the *Plasmodium* circumsporozoite protein- and thrombospondin-related anonymous protein (TRAP)-related protein (CTRP). CTRP is expressed in the *Plasmodium* ookinete, and disruption of this gene leads to reduced motility and failure to invade the mosquito midgut epithelium (81). Furthermore, CTRP is part of a protein family, which includes TgMIC2 and *Plasmodium* TRAP, that is involved in motility and invasion (82). TgME49_209920 is a gene encoding for a putative PAN domain-containing protein with homology to microneme protein (MIC)-4 (MIC4-Like). *Toxoplasma* MIC genes have been shown to play a role in attachment/invasion (83). It is therefore possible that TGME49_259670 and MIC4-Like are involved in the motility and invasion mechanisms of sporozoites.

In summary, genes involved in sporozoite invasion or motility might be good targets for the development of new drugs and vaccines against toxoplasmosis. In addition, the overexpression of genes important for the resistance of sporozoites, such as LEAs, could make the live-attenuated vaccine strains stable and facilitate their production *in vitro*.

## Conclusions and Future Perspectives

Cats serve an important epidemiological role in spreading toxoplasmosis, as they are the only host able to shed oocysts in their feces. Although live-attenuated vaccines can partially protect immunized animals against subsequent infections, full protection is rarely attained. Moreover, vaccines based on mutant strains that are deficient in forming the chronic phase of the parasite cannot be extensively used due to the possibility of reversion to virulent phenotypes. In order to design novel live-attenuated vaccines with known genetic modifications that can be easily stored in the long term, we propose a strategy based on environmentally resistant chronic forms of *Toxoplasma* (oocysts and tissue cysts). For example, by targeting genes that are important for the *in vivo* fitness of the parasite, conversion to the bradyzoite stage and cyst formation, an “oocyst vaccine” could be obtained that elicits a protective immune response while preventing transmission. Although there is a challenge to produce oocysts from such a strain, as the parasite first needs to complete the whole life cycle in cats, recent cutting-edge engineering techniques make it possible to disrupt targeted genes only after reaching the cat’s intestine, when they are no longer needed. Moreover, further improvements and refinement of the groundbreaking systems described by Di Genova et al. (53), Farhat et al. (54) and Humayun et al. (55) might lead to the *in vitro* production of oocysts from such a mutant strain without the need for using mice or cats. Finally, a better understanding of the genes involved in invasion and resistance of sporozoites present in the oocysts could lead to the discovery of valuable drug or vaccine targets that may prevent animals from getting infected and therefore spread the disease to other animals or humans.

Because oocysts are highly resistant and infective, a single-time generation of oocyst would suffice to potentially vaccinate thousands of animals in a short period of time without the need for special storage, significantly facilitating the task. Notwithstanding, it is worth noting that the implementation of this vaccination strategy would face many hurdles along the way. For instance, it would be theoretically possible that a recombination event might take place in the intestine of potentially vaccinated cats harboring other wild-type strains and thus generate parasites with reversion to, or mixture of, wild-type features. This scenario would be highly unlikely to occur in domestic cats with limited, or lack of, outdoor activities, as intestinal stages are only formed in the initial stages after infection and thus vaccination with the recombinant strain would need to happen at a very specific and short span of time, provided they were even previously infected. However, in a more natural setup, for example in outdoor domestic cats or wild felids, it would not be unfeasible to have one or many concurrent infections with other natural strains at the time of vaccination that might elicit this potential undesired situation. Nevertheless, this issue could be easily addressed by first ensuring the negative immunologic status of the animals to be vaccinated (i.e. by serology) before doing so.

Another hindrance would be to perform the vaccination in as many cats as possible, including both domestic and wild felines, within a geographical location. For the former, apart from offering the immunization free of charge, the willingness of each owner needs to be considered. Hence, measurements including educational information and discussion of potential benefits, as well as incentives (for example free serological and coprological analysis, or complimentary regular vaccination/deparasitation treatments) should be implemented. As for wild felids, joint efforts would be required to capture animals, assess their status as mentioned above and perform oral inoculation under sedation before releasing them back into their habitat. Moreover, it is unrealistic to reach all animals, either domestic or wild, outside secluded venues; nevertheless, even vaccination of some animals in a specific location could lead to a significant reduction in the exposure to oocysts of other animals and humans (32), having a significant positive impact in both animal and public health.

## Data Availability Statement

The original contributions presented in the study are included in the article/supplementary material. Further inquiries can be directed to the corresponding author.

## Author Contributions

DA-S and JS have contributed to the conceptualization, writing, and editing of this manuscript. Both authors contributed to the article and approved the submitted version.

## Funding

This study was supported by the National Institutes of Health (1R21AI139387-01) awarded to JS. DA-S was partially supported by the Center for Companion Animal Health (CCAH) at UC Davis with the following grants: 2016-21-F, 2017-11-F, 2018-53-F and 2019-12-F.

## Conflict of Interest

The authors declare that the research was conducted in the absence of any commercial or financial relationships that could be construed as a potential conflict of interest.

## Publisher’s Note

All claims expressed in this article are solely those of the authors and do not necessarily represent those of their affiliated organizations, or those of the publisher, the editors and the reviewers. Any product that may be evaluated in this article, or claim that may be made by its manufacturer, is not guaranteed or endorsed by the publisher.

## References

[1] Chakraborty S Roy S Mistry HU Murthy S George N Bhandari V. Potential Sabotage of Host Cell Physiology by Apicomplexan Parasites for Their Survival Benefits. Front Immunol (2017) 8:1261. doi: 10.3389/fimmu.2017.01261 29081773 PMC5645534

[2] Pinto-Ferreira F Caldart ET Pasquali AKS Mitsuka-Breganó R Freire RL Navarro IT. Patterns of Transmission and Sources of Infection in Outbreaks of Human Toxoplasmosis. Emerg Infect Dis (2019) 25:2177–82. doi: 10.3201/eid2512.181565 PMC6874273 31742524

[3] Mukhopadhyay D Arranz-Solís D Saeij JPJ. Influence of the Host and Parasite Strain on the Immune Response During *Toxoplasma* Infection. Front Cell Infect Microbiol (2020) 10:580425. doi: 10.3389/fcimb.2020.580425 33178630 PMC7593385

[4] Arranz-Solís D Mukhopadhyay D Saeij JJP. *Toxoplasma* Effectors That Affect Pregnancy Outcome. Trends Parasitol (2021) 37:283–95. doi: 10.1016/j.pt.2020.10.013 PMC7954850 33234405

[5] Stelzer S Basso W Benavides Silván J Ortega-Mora LM Maksimov P Gethmann J. *Toxoplasma Gondii* Infection and Toxoplasmosis in Farm Animals: Risk Factors and Economic Impact. Food Waterborne Parasitol (2019) 15:e00037. doi: 10.1016/j.fawpar.2019.e00037 32095611 PMC7033994

[6] Rougier S Montoya JG Peyron F. Lifelong Persistence of *Toxoplasma* Cysts: A Questionable Dogma? Trends Parasitol (2017) 33:93–101. doi: 10.1016/j.pt.2016.10.007 27939103

[7] Dabritz HA Conrad PA. Cats and *Toxoplasma*: Implications for Public Health. Zoonoses Public Health (2010) 57:34–52. doi: 10.1111/j.1863-2378.2009.01273.x 19744306

[8] Freppel W Ferguson DJP Shapiro K Dubey JP Puech P-H Dumètre A. Structure, Composition, and Roles of the *Toxoplasma Gondii* Oocyst and Sporocyst Walls. Cell Surface (2019) 5:100016. doi: 10.1016/j.tcsw.2018.100016 32743133 PMC7389338

[9] Frenkel JK Dubey JP. Effects of Freezing on the Viability of *Toxoplasma* Oocysts. J Parasitol (1973) 59:587. doi: 10.2307/3278803 4711675

[10] Yamaura H. Others. Studies on *Toxoplasma* Oocysts. I. Effects of Low Temperature and Dryness on the Viability of the Oocysts. Kisechugaku Zasshi (1976) 25:80–6.

[11] Kuticic V Wikerhauser T. Studies of the Effect of Various Treatments on the Viability of *Toxoplasma Gondii* Tissue Cysts and Oocysts. Curr Top Microbiol Immunol (1996) 219:261–5. doi: 10.1007/978-3-642-51014-4_23 8791706

[12] Dubey JP. *Toxoplasma Gondii* Oocyst Survival Under Defined Temperatures. J Parasitol (1998) 84:862–5. doi: 10.2307/3284606 9714227

[13] Rousseau A Villena I Dumètre A Escotte-Binet S Favennec L Dubey JP. Evaluation of Propidium Monoazide–Based qPCR to Detect Viable Oocysts of. Toxoplasma Gondii Parasitol Res (2019) 118:999–1010. doi: 10.1007/s00436-019-06220-1 30729299

[14] Dubey JP Miller NL Frenkel JK. The *Toxoplasma Gondii* Oocyst From Cat Feces. J Exp Med (1970) 132:636–62. doi: 10.1084/jem.132.4.636 PMC2138867 4927658

[15] Frenkel JK Dubey JP. Toxoplasmosis and Its Prevention in Cats and Man. J Infect Dis (1972) 126:664–73. doi: 10.1093/infdis/126.6.664 4677084

[16] Ito S Tsunoda K Shimada K Taki T Matsui T. Disinfectant Effects of Several Chemicals Against *Toxoplasma* Oocysts. Nihon Juigaku Zasshi (1975) 37:229–34. doi: 10.1292/jvms1939.37.229 1238847

[17] Wainwright KE Miller MA Barr BC Gardner IA Melli AC Essert T. Chemical Inactivation of *Toxoplasma Gondii* Oocysts in Water. J Parasitol (2007) 93:925–31. doi: 10.1645/GE-1063R.1 17918377

[18] Villegas EN Augustine SAJ Villegas LF Ware MW See MJ Lindquist HDA. Using Quantitative Reverse Transcriptase PCR and Cell Culture Plaque Assays to Determine Resistance of *Toxoplasma Gondii* Oocysts to Chemical Sanitizers. J Microbiol Methods (2010) 81:219–25. doi: 10.1016/j.mimet.2010.03.023 20385175

[19] Barrs VR Martin P Beatty JA. Antemortem Diagnosis and Treatment of Toxoplasmosis in Two Cats on Cyclosporin Therapy. Aust Vet J (2006) 84:30–5. doi: 10.1111/j.1751-0813.2006.tb13119.x 16498831

[20] Montazeri M Mikaeili Galeh T Moosazadeh M Sarvi S Dodangeh S Javidnia J. The Global Serological Prevalence of *Toxoplasma Gondii* in Felids During the Last Five Decades (1967–2017): A Systematic Review and Meta-Analysis. Parasit Vectors (2020) 13:1–10. doi: 10.1186/s13071-020-3954-1 32066495 PMC7026947

[21] Hatam-Nahavandi K Calero-Bernal R Rahimi MT Pagheh AS Zarean M Dezhkam A. *Toxoplasma Gondii* Infection in Domestic and Wild Felids as Public Health Concerns: A Systematic Review and Meta-Analysis. Sci Rep (2021) 11:9509. doi: 10.1038/s41598-021-89031-8 33947922 PMC8097069

[22] Vollaire MR Radecki SV Lappin MR. Seroprevalence of *Toxoplasma Gondii* Antibodies in Clinically Ill Cats in the United States. Am J Vet Res (2005) 66:874–7. doi: 10.2460/ajvr.2005.66.874 15934615

[23] Dabritz HA Gardner IA Miller MA Lappin MR Atwill ER Packham AE. Evaluation of Two *Toxoplasma Gondii* Serologic Tests Used in a Serosurvey of Domestic Cats in California. J Parasitol (2007) 93:806–16. doi: 10.1645/GE-996R.1 17918359

[24] Gondim LFP Mineo JR Schares G. Importance of Serological Cross-Reactivity Among *Toxoplasma Gondii*, *Hammondia* Spp., *Neospora* Spp., *Sarcocystis* Spp. And *Besnoitia Besnoiti* . Parasitology (2017) 144:851–68. doi: 10.1017/S0031182017000063 PMC5471829 28241894

[25] Arranz-Solís D Cordeiro C Young LH Dardé ML Commodaro AG Grigg ME. Serotyping of *Toxoplasma Gondii* Infection Using Peptide Membrane Arrays. Front Cell Infect Microbiol (2019) 9:408. doi: 10.3389/fcimb.2019.00408 31850240 PMC6895565

[26] Arranz-Solís D Carvalheiro CG Zhang ER Grigg ME Saeij JPJ. *Toxoplasma* GRA Peptide-Specific Serologic Fingerprints Discriminate Among Major Strains Causing Toxoplasmosis. Front Cell Infect Microbiol (2021) 11:621738. doi: 10.3389/fcimb.2021.621738 33680990 PMC7935526

[27] Dubey JP Frenkel JK. Immunity to Feline Toxoplasmosis: Modification by Administration of Corticosteroids. Vet Pathol (1974) 11:350–79. doi: 10.1177/030098587401100407 4460329

[28] Zhu S Shapiro K VanWormer E. Dynamics and Epidemiology of *Toxoplasma Gondii* Oocyst Shedding in Domestic and Wild Felids. Transbound Emerg Dis (2021). doi: 10.1111/tbed.14197 34153160

[29] Dubey JP. Effect of Immunization of Cats With *Isospora Felis* and BCG on Immunity to Reexcretion of *Toxoplasma Gondii* Oocysts. J Protozool (1978) 25:380–2. doi: 10.1111/j.1550-7408.1978.tb03909.x 364044

[30] Dubey JP Lappin MR Thulliez P. Long-Term Antibody Responses of Cats Fed *Toxoplasma Gondii* Tissue Cysts. J Parasitol (1995) 81:887–93. doi: 10.2307/3284035 8544059

[31] Zulpo DL Sammi AS dos Santos JR Sasse JP Martins TA Minutti AF. *Toxoplasma Gondii*: A Study of Oocyst Re-Shedding in Domestic Cats. Vet Parasitol (2018) 249:17–20. doi: 10.1016/j.vetpar.2017.10.021 29279081

[32] Bonačić Marinović AA Opsteegh M Deng H Suijkerbuijk AWM van Gils PF van der Giessen J. Prospects of Toxoplasmosis Control by Cat Vaccination. Epidemics (2019) 30:100380. doi: 10.1016/j.epidem.2019.100380 31926434

[33] Innes EA Bartley PM Maley S Katzer F Buxton D. Veterinary Vaccines Against. Toxoplasma Gondii Mem Inst Oswaldo Cruz (2009) 104:246–51. doi: 10.1590/s0074-02762009000200018 19430650

[34] Buxton D Innes EA. A Commercial Vaccine for Ovine Toxoplasmosis. Parasitology (1995) 110 Suppl:S11–6. doi: 10.1017/s003118200000144x 7784124

[35] Elmore SA Jones JL Conrad PA Patton S Lindsay DS Dubey JP. *Toxoplasma Gondii*: Epidemiology, Feline Clinical Aspects, and Prevention. Trends Parasitol (2010) 26:190–6. doi: 10.1016/j.pt.2010.01.009 20202907

[36] Pfefferkorn ERR Pfefferkorn LGC Characterization P. *Toxoplasma Gondii*: Isolation and Preliminary Characterization of Temperature-Sensitive Mutants. Exp Parasitol (1976) 39:365–76. doi: 10.1016/0014-4894(76)90040-0 1269580

[37] Frenkel JK Pfefferkorn ER Smith DD Fishback JL. Prospective Vaccine Prepared From a New Mutant of *Toxoplasma Gondii* for Use in Cats. Am J Vet Res (1991) 52:759–63. 1854103

[38] Freyre A Choromanski L Fishback JL Popiel I. Immunization of Cats With Tissue Cysts, Bradyzoites, and Tachyzoites of the T-263 Strain of. Toxoplasma Gondii J Parasitol (1993) 79:716–9. doi: 10.2307/3283610 8410543

[39] Dubey JP. Schizogony and Gametogony of Oocyst-Deficient T-263 Strain of *Toxoplasma Gondii* . Vet Parasitol (2017) 245:160–2. doi: 10.1016/j.vetpar.2017.05.024 28624132

[40] Mateus-Pinilla NE Dubey JP Choromanski L Weigel RM. A Field Trial of the Effectiveness of a Feline *Toxoplasma Gondii* Vaccine in Reducing T. Gondii exposure Swine J Parasitol (1999) 85:855–60. 10577720

[41] Dubey JP. Strategies to Reduce Transmission of *Toxoplasma Gondii* to Animals and Humans. Vet Parasitol (1996) 64:65–70. doi: 10.1016/0304-4017(96)00961-2 8893464

[42] Omata Y Aihara Y Kanda M Saito A Igarashi I Suzuki N. *Toxoplasma Gondii*: Experimental Infection in Cats Vaccinated With ^60^Co-Irradiated Tachyzoites. Vet Parasitol (1996) 65:173–83. doi: 10.1016/S0304-4017(96)00973-9 8983144

[43] Ramakrishnan C Maier S Walker RA Rehrauer H Joekel DE Winiger RR. An Experimental Genetically Attenuated Live Vaccine to Prevent Transmission of *Toxoplasma Gondii* by Cats. Sci Rep (2019) 9:1474. doi: 10.1038/s41598-018-37671-8 30728393 PMC6365665

[44] Le Roux D Djokic V Morisse S Chauvin C Doré V Lagrée A-C. Evaluation of Immunogenicity and Protection of the Mic1-3 Knockout *Toxoplasma Gondii* Live Attenuated Strain in the Feline Host. Vaccine (2020) 38:1457–66. doi: 10.1016/j.vaccine.2019.11.076 31864855

[45] Behnke MS Zhang TP Dubey JP Sibley LD. *Toxoplasma Gondii* Merozoite Gene Expression Analysis With Comparison to the Life Cycle Discloses a Unique Expression State During Enteric Development. BMC Genomics (2014) 15:350. doi: 10.1186/1471-2164-15-350 24885521 PMC4035076

[46] Tomita T Bzik DJ Ma YF Fox BA Markillie LM Taylor RC. The *Toxoplasma Gondii* Cyst Wall Protein CST1 is Critical for Cyst Wall Integrity and Promotes Bradyzoite Persistence. PloS Pathog (2013) 9:e1003823. doi: 10.1371/journal.ppat.1003823 24385904 PMC3873430

[47] Zeiner GM Boothroyd JC Liu L Hirschberg CB Koshy AA Caffaro CE. A Nucleotide Sugar Transporter Involved in Glycosylation of the *Toxoplasma* Tissue Cyst Wall Is Required for Efficient Persistence of Bradyzoites. PloS Pathog (2013) 9:e1003331. doi: 10.1371/journal.ppat.1003331 23658519 PMC3642066

[48] Walker R Gissot M Croken MM Huot L Hot D Kim K. The *Toxoplasma* Nuclear Factor TgAP2XI-4 Controls Bradyzoite Gene Expression and Cyst Formation. Mol Microbiol (2013) 87:641–55. doi: 10.1111/mmi.12121 PMC3556193 23240624

[49] Waldman BS Schwarz D Wadsworth MH 2nd Saeij JP Shalek AK Lourido S. Identification of a Master Regulator of Differentiation in *Toxoplasma* . Cell (2020) 180:359–372.e16. doi: 10.1016/j.cell.2019.12.013 31955846 PMC6978799

[50] Drewry LL Jones NG Wang Q Onken MD Miller MJ Sibley LD. The Secreted Kinase ROP17 Promotes *Toxoplasma Gondii* Dissemination by Hijacking Monocyte Tissue Migration. Nat Microbiol (2019) 4:1951–63. doi: 10.1038/s41564-019-0504-8 PMC6814536 31332383

[51] Sangaré LO Ólafsson EB Wang Y Yang N Julien L Camejo A. *In Vivo* CRISPR Screen Identifies TgWIP as a *Toxoplasma* Modulator of Dendritic Cell Migration. Cell Host Microbe (2019) 26:478–492.e8. doi: 10.1016/j.chom.2019.09.008 31600500 PMC7060943

[52] Franco M Panas MW Marino ND Lee M-CW Buchholz KR Kelly FD. A Novel Secreted Protein, MYR1, Is Central to *Toxoplasma* ‘S Manipulation of Host Cells. MBio (2016) 7:1–16. doi: 10.1128/mbio.02231-15 PMC4742717 26838724

[53] Martorelli Di Genova B Wilson SK Dubey JP Knoll LJ. Intestinal Delta-6-Desaturase Activity Determines Host Range for *Toxoplasma* Sexual Reproduction. PloS Biol (2019) 17:e3000364. doi: 10.1371/journal.pbio.3000364 31430281 PMC6701743

[54] Farhat DC Swale C Dard C Cannella D Ortet P Barakat M. A MORC-Driven Transcriptional Switch Controls *Toxoplasma* Developmental Trajectories and Sexual Commitment. Nat Microbiol (2020) 5:570–83. doi: 10.1038/s41564-020-0674-4 PMC7104380 32094587

[55] Humayun M Ayuso JM Park KY Martorelli Di Genova B Skala MC Kerr SC. Innate Immune Cell Response to Host-Parasite Interaction in a Human Intestinal Tissue Microphysiological System. Sci Adv (2022) 8:eabm8012. doi: 10.1126/sciadv.abm8012 35544643 PMC9075809

[56] Freyre A Dubey JP Smith DD Frenkel JK. Oocyst-Induced *Toxoplasma Gondii* Infections in Cats. J Parasitol (1989) 75:750–5. doi: 10.2307/3283060 2795377

[57] Dubey JP. Infectivity and Pathogenicity of *Toxoplasma Gondii* Oocysts for Cats. J Parasitol (1996) 82:957. doi: 10.2307/3284206 8973406

[58] Dubey JP Frenkel JK. Cyst-Induced Toxoplasmosis in Cats*. J Protozool (2007) 19:155–77. doi: 10.1111/j.1550-7408.1972.tb03431.x 5008846

[59] Dubey JP. Comparative Infectivity of Oocysts and Bradyzoites of *Toxoplasma Gondii* for Intermediate (Mice) and Definitive (Cats) Hosts. Vet Parasitol (2006) 140:69–75. doi: 10.1016/j.vetpar.2006.03.018 16647212

[60] van Poppel NFJJ Welagen J Duisters RFJJJJ Vermeulen AN Schaap D. Tight Control of Transcription in *Toxoplasma Gondii* Using an Alternative Tet Repressor. Int J Parasitol (2006) 36:443–52. doi: 10.1016/j.ijpara.2006.01.005 16516216

[61] Etheridge RDD Alaganan A Tang K Lou HJJ Turk BEE Sibley LDD. The *Toxoplasma* Pseudokinase ROP5 Forms Complexes With ROP18 and ROP17 Kinases That Synergize to Control Acute Virulence in Mice. Cell Host Microbe (2014) 15:537–50. doi: 10.1016/j.chom.2014.04.002 PMC4086214 24832449

[62] Banaszynski LA Chen L-C Maynard-Smith LA Ooi AGL Wandless TJ. A Rapid, Reversible, and Tunable Method to Regulate Protein Function in Living Cells Using Synthetic Small Molecules. Cell (2006) 126:995–1004. doi: 10.1016/j.cell.2006.07.025 16959577 PMC3290523

[63] Paredes-Santos TC Martins-Duarte ES Vitor RWAA de Souza W Attias M Vommaro RC. Spontaneous Cystogenesis *In Vitro* of a Brazilian Strain of *Toxoplasma Gondii* . Parasitol Int (2013) 62:181–8. doi: 10.1016/j.parint.2012.12.003 23269201

[64] McPhillie M Zhou Y El Bissati K Dubey J Lorenzi H Capper M. New Paradigms for Understanding and Step Changes in Treating Active and Chronic, Persistent Apicomplexan Infections. Sci Rep (2016) 6:29179. doi: 10.1038/srep29179 27412848 PMC4944145

[65] Christiansen C Maus D Hoppenz E Murillo-León M Hoffmann T Scholz J. *In Vitro* Maturation of *Toxoplasma Gondii* Bradyzoites in Human Myotubes and Their Metabolomic Characterization. Nat Commun (2022) 13:1168. doi: 10.1038/s41467-022-28730-w 35246532 PMC8897399

[66] Douglas T. The Dual-Use Problem, Scientific Isolationism and the Division of Moral Labour. Monash Bioeth Rev (2014) 32:86–105. doi: 10.1007/s40592-014-0004-9 25434066 PMC4210745

[67] Harb OS Kissinger JC Roos DS. “ToxoDB: The Functional Genomic Resource for Toxoplasma and Related Organisms.,”. In: Toxoplasma Gondii. Elsevier (2020). p. 1021–41. Available at: https://www.sciencedirect.com/science/article/pii/B9780128150412000232. 10.1007/978-1-4939-9857-9_2 31758445

[68] Fritz HM Bowyer PW Bogyo M Conrad PA Boothroyd JC. Proteomic Analysis of Fractionated *Toxoplasma* Oocysts Reveals Clues to Their Environmental Resistance. PloS One (2012) 7:e29955. doi: 10.1371/journal.pone.0029955 22279555 PMC3261165

[69] Possenti A Fratini F Fantozzi L Pozio E Dubey JP Ponzi M. Global Proteomic Analysis of the Oocyst/Sporozoite of *Toxoplasma Gondii* Reveals Commitment to a Host-Independent Lifestyle. BMC Genomics (2013) 14:183. doi: 10.1186/1471-2164-14-183 23496850 PMC3616887

[70] Fritz HM Buchholz KR Chen X Durbin-Johnson B Rocke DM Conrad PA. Transcriptomic Analysis of *Toxoplasma* Development Reveals Many Novel Functions and Structures Specific to Sporozoites and Oocysts. PloS One (2012) 7:e29998. doi: 10.1371/journal.pone.0029998 22347997 PMC3278417

[71] Hehl AB Basso WU Lippuner C Ramakrishnan C Okoniewski M Walker RA. Asexual Expansion of Toxoplasma Gondii Merozoites is Distinct From Tachyzoites and Entails Expression of non-Overlapping Gene Families to Attach, Invade, and Replicate Within Feline Enterocytes. BMC Genomics (2015) 16:66. doi: 10.1186/s12864-015-1225-x 25757795 PMC4340605

[72] Hincha DK Thalhammer A. LEA Proteins: IDPs With Versatile Functions in Cellular Dehydration Tolerance. Biochem Soc Trans (2012) 40:1000–3. doi: 10.1042/BST20120109 22988854

[73] Hill D Coss C Dubey JP Wroblewski K Sautter M Hosten T. Identification of a Sporozoite-Specific Antigen From *Toxoplasma Gondii* . J Parasitol (2011) 97:328–37. doi: 10.1645/GE-2782.1 PMC3684278 21506817

[74] Sancar A. Structure and Function of DNA Photolyase. Biochemistry (1994) 33:2–9. doi: 10.1021/bi00167a001 8286340

[75] Sancar A. Mechanisms of DNA Repair by Photolyase and Excision Nuclease (Nobel Lecture). Angew Chem Int Ed Engl (2016) 55:8502–27. doi: 10.1002/anie.201601524 27337655

[76] Holmgren A. Hydrogen Donor System for *Escherichia Coli* Ribonucleoside-Diphosphate Reductase Dependent Upon Glutathione. Proc Natl Acad Sci USA (1976) 73:2275–9. doi: 10.1073/pnas.73.7.2275 PMC430527 7783

[77] Ogata FT Branco V Vale FF Coppo L. Glutaredoxin: Discovery, Redox Defense and Much More. Redox Biol (2021) 43:101975. doi: 10.1016/j.redox.2021.101975 33932870 PMC8102999

[78] Crawford J Lamb E Wasmuth J Grujic O Grigg ME Boulanger MJ. Structural and Functional Characterization of SporoSAG: A SAG2-Related Surface Antigen From *Toxoplasma Gondii* . J Biol Chem (2010) 285:12063–70. doi: 10.1074/jbc.M109.054866 PMC2852944 20164173

[79] Poukchanski A Fritz HM Tonkin ML Treeck M Boulanger MJ Boothroyd JC. *Toxoplasma Gondii* Sporozoites Invade Host Cells Using Two Novel Paralogues of RON2 and AMA1. PloS One (2013) 8:e70637. doi: 10.1371/journal.pone.0070637 23940612 PMC3734201

[80] Parker ML Penarete-Vargas DM Hamilton PT Guérin A Dubey JP Perlman SJ. Dissecting the Interface Between Apicomplexan Parasite and Host Cell: Insights From a Divergent AMA–RON2 Pair. Proc Natl Acad Sci U.S.A. (2016) 113:398–403. doi: 10.1073/pnas.1515898113 26712012 PMC4720339

[81] Dessens JT Beetsma AL Dimopoulos G Wengelnik K Crisanti A Kafatos FC. CTRP is Essential for Mosquito Infection by Malaria Ookinetes. EMBO J (1999) 18:6221–7. doi: 10.1093/emboj/18.22.6221 PMC1171685 10562534

[82] Baum J Richard D Healer J Rug M Krnajski Z Gilberger TW. A Conserved Molecular Motor Drives Cell Invasion and Gliding Motility Across Malaria Life Cycle Stages and Other Apicomplexan Parasites. J Biol Chem (2006) 281:5197–208. doi: 10.1074/jbc.M509807200 16321976

[83] Bisio H Soldati-Favre D. Signaling Cascades Governing Entry Into and Exit From Host Cells by *Toxoplasma Gondii* . Annu Rev Microbiol (2019) 73:579–99. doi: 10.1146/annurev-micro-020518-120235 31500539

